# New Jersey’s Attempted Legislation

**Published:** 1914-06

**Authors:** 


### New Jersey's Attempted Legislation.

It is expected that, in considering any question of great moment to the public as a whole, there will appear many attempts to hold the matter in ridicule or to thwart its purposes. Such was the attitude recently taken by the Assembly of the State of New Jersey in passing the following bill, which, however, was defeated by the Senate:

"The teaching of sex hygiene or sexology, and the distribution of any books or pamphlets in which such subjects are treated or discussed, in any school receiving any portion of the moneys appropriated for the support of public schools, is hereby prohibited."

